# Percutaneous endoscopic gastrostomy with Funada-style gastropexy greatly reduces the risk of peristomal infection

**DOI:** 10.1093/gastro/gou086

**Published:** 2015-01-06

**Authors:** Naoki Okumura, Naoko Tsuji, Nobuto Ozaki, Nozomu Matsumoto, Takehisa Takaba, Masanori Kawasaki, Takafumi Tomita, Yasuko Umehara, Satoko Taniike, Masashi Kono, Masatoshi Kudo

**Affiliations:** ^1^Department of Gastroenterology, Sakai Hospital, Kinki University Faculty of Medicine, Sakai, Japan; ^2^Department of Gastroenterology and Hepatology, Kinki University Faculty of Medicine, Osaka-sayama, Japan

**Keywords:** percutaneous endoscopic gastrostomy, gastropexy, peristomal infection

## Abstract

**Background and aims:** Peristomal wound infections are common complications of percutaneous endoscopic gastrostomy (PEG). The Funada-style gastropexy device has two parallel needles with a wire loop and suture thread, and was developed about 20 years ago in Japan. This kit has allowed us to perform dual gastropexy very easily; PEG with gastropexy has become a very popular technique in Japan. The present study aimed to compare the advantages and disadvantages of PEG with the gastropexy technique with the standard ‘pull' method.

**Methods:** We retrospectively reviewed 182 consecutive, non-randomized patients undergoing PEG in our hospital, and a comparative analysis was made between the gastropexy (87 patients) and non-gastropexy (95 patients) groups.

**Results:** The rates of patients having erythema (11.6% *vs.* 47.9%; *P < *0.001), exudates (2.3% *vs.* 14.9%; *P < *0.01) and infection (0% *vs.* 6.4%; *P = *0.01) in the peristomal area were lower in the gastropexy than in the non-gastropexy group. The rate of minor bleeding from the peristomal area was higher in the gastropexy than in the non-gastropexy group (12.8% *vs.* 2.1%; *P < *0.01), but no patient required a blood transfusion. Mean procedure time was longer in the gastropexy group than in the non-gastropexy group (31 *vs.* 24 min; *P < *0.001). The 30-day mortality rates were 4.7% and 5.3% respectively, and these deaths were not related to the gastrostomy procedure.

**Conclusion:** PEG with gastropexy markedly reduces peristomal inflammation. Although minor bleeding and a longer procedure time were disadvantages, there were no severe complications. The findings suggested that PEG with Funada-style gastropexy was a safe and feasible method for reducing early complications of PEG.

## Introduction

Percutaneous endoscopic gastrostomy (PEG) is currently the standard method for enteral nutrition in patients with swallowing disorders, and the most widely used technique is the pull- or pull-through method described by Ponsky and Gauderer [1]. Fistula infections are a common early complication, and are believed to be caused by bacterial infection of the tube when it passes through the oropharynx. The introducer-PEG that is placed transabdominally into the stomach was first reported about 30 years ago [2], but it did not become widely used because it was technically difficult and associated with many complications; however, a Japanese surgeon invented a new gastropexy kit in 1991 [3]. This kit allowed us to perform dual gastropexy very easily and introducer PEG with gastropexy has become a common technique in Japan. The aim of our study was to assess the advantages and disadvantages of PEG with the gastropexy technique, compared with the standard pull method.

## Patients and methods

### Patients

**Figure 1.**
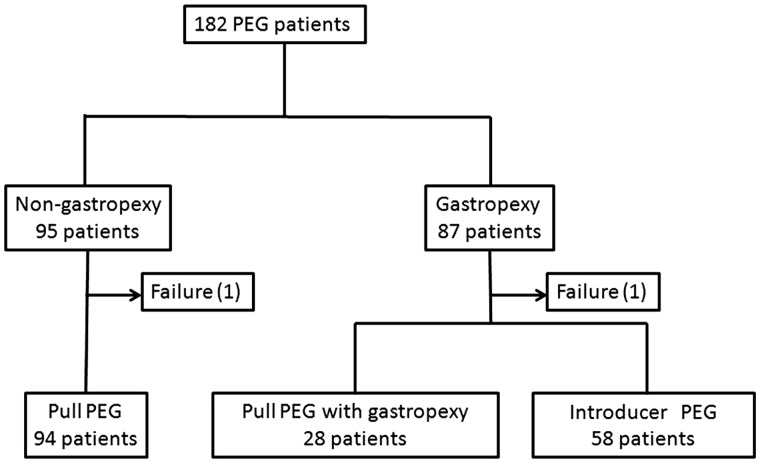
Flow diagram summarizing patients treated with different PEG methods.

We retrospectively reviewed 182 consecutive patients undergoing PEG in our hospital between 2003 and 2009. There were 95 patients in the standard ‘pull non-gastropexy' group and 87 patients in the gastropexy group. The gastropexy group consisted of 28 patients undergoing pull-PEG with gastropexy and 58 patients receiving modified introducer-PEG (Figure 1). The anterior wall of the middle-to-lower body of the stomach was chosen as the PEG site. We compared the peristomal inflammation, bleeding, overall procedure times, and short-term prognoses of the two groups. All patients received prophylactic antibiotics. The diameters of erythema were measured and indulation, exudates, ulcer, and bleeding were recorded by nurses in charge, according to the clinical pathway of our hospital and, if infection was suspected, a Polaroid photograph of the stomal site was attached to the patient’s records. We diagnosed peristomal infection based on a combination of erythema, exudates, and induration, development of pus, or focal peritonitis. All bleedings—intra-operative and post-operative, including very minor ones controlled by compresses—were recorded.

Most of the procedures were conducted by fellows of our division. Overall procedure durations were timed from skin disinfection before procedure until fixation of the feeding tube and re-sterilization of the skin after removing the endoscope. All patients received an abdominal computed tomography (CT) scan before the procedure. The study was conducted in accordance with the principles of the Helsinki Declaration and was approved by the local ethics committee of Kinki University Faculty of Medicine.

### Pull method (Ponsky technique)

In this technique, the stomach was inflamed by introducing air via an endoscope. After transillumination, a small incision is made and a needle is inserted through the abdominal wall into the stomach, a guide wire is passed through the needle, grasped endoscopically and withdrawn through the mouth. A 20 Fr gastrostomy tube (Bard PEG Kit Safety system, MEDICON INC, Japan) is affixed to the guide wire before being pulled through the oesophagus into the stomach and out of the abdominal wall.

### Dual gastropexy

**Figure 2.**
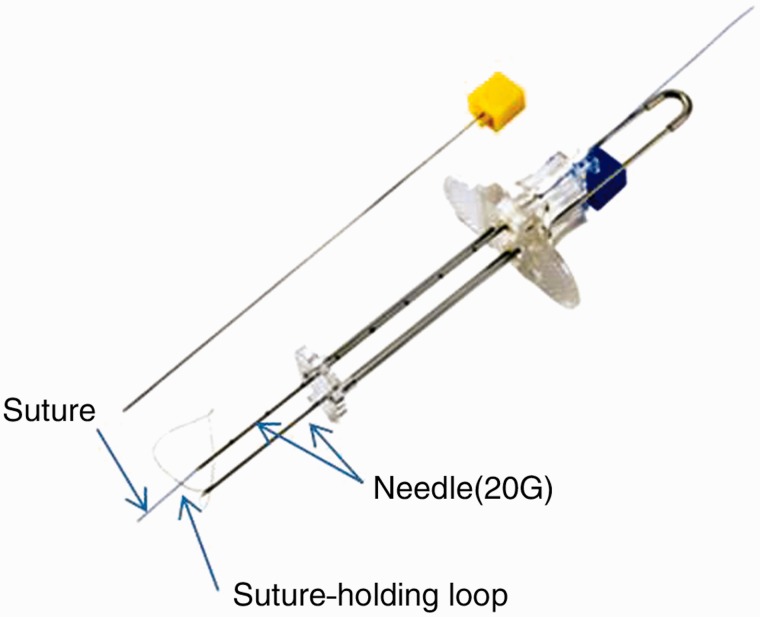
Funada-style gastropexy device. By courtesy of the Create Medic Company.

**Figure 3.**
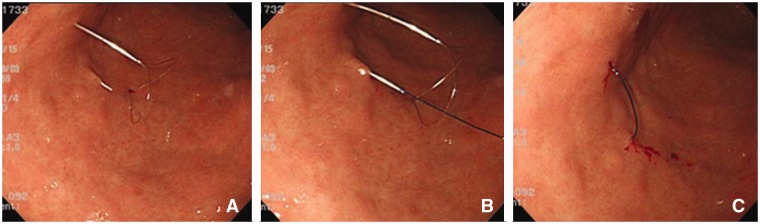
Endoscopic view of Funada-type gastropexy. Puncture of two parallel needles and forming a loop to hold the suture (A). The distal end of the suture from one straight needle passes through the loop wire from the other needle (B). The loop is placed back in the needle and the device is pulled out of the body (C). The freed suture is knotted.

The Funada-style gastropexy device has two parallel, 20-gauge needles and a suture-holding loop (Figure 2). After transillumination, the two parallel needles penetrate both the abdominal and anterior gastric walls. The suture-holding loop is inserted through the first needle. The suture thread is inserted through the second needle and grasped with the snare. The needles are withdrawn and the suture thread is tied on the abdominal wall (Figure 3). In the same way, a second gastropexy is made about 2 cm away.

### Modified introducer method

**Figure 4.**
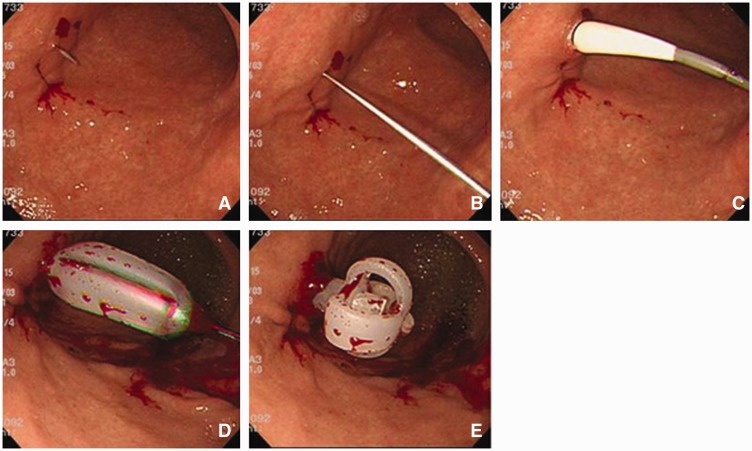
Endoscopic view of modified introducer methods. Puncture the stomach with an 18-gauge needle in the center of two sutures (A), insert the guide wire through the needle (B), insert the dilator over the guide wire (C), insert a 24 Fr bumper-type gastrostomy tube (D), finely open the bumper (E).

After dual gastropexy, a skin incision about 1.5 cm in length was made between the two gastropexy sites. We punctured the stomach with an 18-gauge needle, inserted the guide wire through the needle, inserted the dilator over the guide wire, removed the dilator, and finally directly inserted a 24 Fr bumper-type gastrostomy tube (Ideal PEG kit, Olympus, Tokyo, Japan or Endovive Seldinger kit, Boston Scientific, Japan) from the abdominal wall (Figure 4). The suture thread was removed 1–2 weeks later.

### Statistics

Statistical analysis was performed using the χ^2^ test and Fisher’s test for categorical data and the Mann-Whitney *U* test for continuous data. IBM SPSS 17.0 software was used, and significance was accepted at a *P*-value of less than 0.05.

## Results

### Non-gastropexy *vs.* gastropexy

The success rates in both groups were 99%. In one patient from each group, transillumination of light at appropriate sites was not possible. There were no significant differences between the non-gastropexy and gastropexy groups with respect to patients' clinical backgrounds, i.e. age and gender distributions, albumin and body mass index (BMI), and basic illnesses (Table 1).

**Table 1.** Demographics and baseline characteristics of patients

	Non-gastropexy (*n* = 94)	Gastropexy (*n* = 86)	*P*-value
Mean age, years (range)	69.7 (30–96)	69.9 (33–90)	0.62
Male, *n* (%)	55 (58.5)	46 (53.5)	0.49
Mean albumin, g/dL (range)	3.3 (2.1–4.8)	3.4 (2.1–4.6)	0.10
Mean BMI, kg/m^2^ (range)	19.3 (11.7–27.4)	18.4 (11.0–29.6)	0.18
Underlying disease, *n* (%)			0.93
CNS disorders	55 (58.5)	53 (61.6)	
Cerebrovascular disorders	22 (23.4)	19 (22.1)	
Tumour	5 (5.3)	3 (3.5)	
Others	12 (12.8)	11 (12.8)	

BMI = body mass index; CNS = central nervous system

The rate of patients with erythema in the peristomal area in the gastropexy group was 11.6%—significantly lower than that of the non-gastropexy group (47.9%; *P < *0.001). The rate for exudate was only 2.3%, compared with 14.9% in the non-gastropexy group (*P* < 0.01). No patient had ulcer formation or focal peritonitis in the gastropexy group. Six patients (6.4%) in non-gastropexy group were diagnosed with peristomal infection, while none was diagnosed with either perisotmal infection or focal peritonitis in the gastropexy group. All patients with peristomal infection received antibiotics. In addition, two patients with focal peritonitis received parenteral nutrition until the peritonitis improved. No patients needed surgical procedures. The rate of bleeding from the peristomal area in the gastropexy group was significantly higher than that in the non-gastropexy group (12.8% *vs.* 2.1%; *P < *0.01), but no patients required a blood transfusion. All post-operative bleeding occurred within three days. The 30-day mortality rates were 4.7% and 5.3%, respectively, and these deaths were unrelated to the gastrostomy procedure. The mean procedure time in the gastropexy group was significantly longer than that in the non-gastropexy group (31 *vs.* 24 minutes; *P < *0.001) (Table 2).

**Table 2.** Comparison of clinical outcomes between non-gastropexy and gastropexy

	Non-gastropexy (*n* = 94)	Gastropexy (*n* = 86)	*P*-value
Peristomal inflammation, *n* (%)			
Erythema	45 (47.9)	10 (11.6)	<0.001
Exudate	14 (14.9)	2 (2.3)	<0.01
Ulcer	2 (2.1)	0	0.51
Focal peritonitis	2 (2.1)	0	0.51
Peristomal infection*, *n* (%)	6 (6.4)	0	0.01
Bleeding, *n* (%)	2 (2.1)	11 (12.8)	<0.01
Mean procedure duration, minutes (range)	23 (6–77)	31 (14–72)	<0.001
30-day mortality, *n* (%)	5 (5.3)	4 (4.7)	0.89

*Combination of erythema, indulation, pus discharge or peritonitis

### Pull-PEG *vs.* gastropexy pull-PEG

To assess the gastropexy effect more rigorously, we compared 28 gastropexy pull-PEG patients and 94 pull-PEG patients (Table 3). The rate of erythema in the gastropexy pull-PEG group was significantly lower than that in the pull-PEG group (10.7% *vs.* 47.9%; *P < *0.001). No patients in the gastropexy pull-PEG group showed peristomal infection. Mean overall procedure time in gastropexy group was significantly longer than that in the standard pull methods (29 *vs.* 23 minutes; *P < *0.001).

**Table 3.** Comparison of clinical outcomes between pull-PEG and agstropexy pull-PEG

	Pull-PEG (*n* = 94)	Gastropexy pull-PEG (*n* = 28)	*P*-value
Peristomal inflammation, *n* (%)			
Erythema	45 (47.9)	3 (10.7)	<0.001
Exudate	14 (14.9)	1 (3.6)	0.09
Ulcer	2 (2.1)	0	0.59
Focal peritonitis	2 (2.1)	0	0.59
Peristomal infection, *n* (%)	6 (6.4)	0	0.20
Bleeding, *n* (%)	2 (2.1)	2 (7.1)	0.22
Mean procedure duration, minutes (range)	23 (6–77)	29 (14–72)	<0.001
30-day mortality, *n* (%)	5 (5.3)	1 (3.6)	0.58

PEG = percutaneous endoscopic gastrostomy

### Pull-PEG *vs.* introducer PEG

To assess the different methods, we compare the pull-PEG and introducer-PEG groups (Table 4). Peristomal inflammation and infection was lower in the introducer-PEG group, while the rate of bleeding was significantly higher and mean procedure time was 9 minutes longer.

**Table 4.** Comparison of clinical outcomes between pull-PEG and modified introducer-PEG

	Pull-PEG (*n* = 94)	Modified introducer-PEG (*n* = 58)	*P*-value
Peristomal inflammation, *n* (%)			
Erythema	45 (47.9)	7 (12.1)	<0.001
Exudate	14 (14.9)	1 (1.7)	<0.01
Ulcer	2 (2.1)	0	0.38
Focal peritonitis	2 (2.1)	0	0.38
Peristomal infection, *n* (%)	6 (6.4)	0	0.05
Bleeding, *n* (%)	2 (2.1)	8 (13.8)	<0.01
Mean procedure duration, minutes (range)	23 (6–77)	32 (17–64)	<0.001
30-day mortality, *n* (%)	5 (5.3)	3 (5.2)	0.63

PEG = percutaneous endoscopic gastrostomy

## Discussion

Gastropexy is mainly used by radiologists to perform direct percutaneous gastrostomy under fluoroscopic guidance [4-6]. T-fastener kits are the most commonly used accessories for gastropexy [7]. The metal T-bars are attached to a suture, with a straight surgical needle attached to the other end. The number of T-fasteners used varies between one and four.

Russell *et al.* first reported percutaneous gastrostomy without gastropexy under endoscopic guidance (introducer method) in 1984 [2]. In his original method, the stomach was punctured using an 18-gauge needle percutaneously, a guide wire was passed through the needle into the stomach, and then the needle was removed. A 16 Fr dilator-peel-away sheath set was passed over the guide wire, and the dilator and guide wire were then removed. A 14 Fr Foley catheter was advanced through the peel-away sheath, after which the sheath was removed.

In 1987, Hashiba reported introducer-PEG with gastropexy [8]. After 2000, several studies on introducer-PEG with gastropexy were reported [9–12]; it has been reported that introducer methods are superior in preventing infection. In patients with head and neck cancers, introducer methods are particularly important prophylactically, to prevent wound infection and cancer metastasis [13, 14].

The reason why introducer methods did not become popular seems to be the complexity of gastropexy. Many PEG-indicated patients are in poor general condition, and the length of the procedure is very important for such patients. The Ponsky (pull) technique is a relatively easy technique for endoscopists, not requiring much time to perform gastrostomy. The main factor referring to the duration of the procedure seems to be finding an appropriate puncture site.

In Japan, Funada invented the gastropexy kit about 20 years ago [3]. The Funada-style gastropexy device has two parallel 20-gauge needles with a wire loop and suture thread (Funada-style Loop Gastropexy Device, Create Medic, Kanagawa, Japan). We think the Funada-style gastropexy and T-bar technique may be almost the same in terms of infection rates and efficacy points, but we believe that Funada-style gastropexy is superior at several pointsin several respects. With the Funada-style procedure, we can fix two points by one operation, using surgical sutures, while with the T-bar technique, we had to fix one by one, and in addition stoppers are necessary on the surface of an abdominal wall. Suture thread is harmless to the human body, while the T-bar technique uses a metallic anchor that may cause foreign-body reaction. We can carry out numerous gastropexies using the Funada technique, requiring only extra suturing thread, as opposed to a quantity of T-bars. Nothing remains in the body when sutures are removed, whereas metallic anchors remain in the gastrointestinal tract when T-bars are removed, and there was a case report of pneumoperitoneum from an eroded T-fastener [15].

Dormann *et al.* reported 46 cases of PEG with gastropexy. They employed the Funada-style device for gastropexy. They used an introducer technique and the gastrostomy tube was the 13 Fr balloon type [9]. The diameter of the initial gastrostomy tube was reportedly 12–19 Fr in a percutaneous radiological gastrostomy or introducer-PEG, being smaller than in pull-PEG (more than 20 Fr). Inoue reported the first modified introducer method in 2002 [16]. In the modified introducer method, a large-bore tube can be initially inserted, as in the pull-PEG methods. After modified introducer-PEG kits became commercially available in Japan, modified introducer-PEG with Funada-style gastropexy became a widely used technique.

Gastropexy has advantages and disadvantages: the main advantage is the reduction of peristomal infection and inflammation, as our study and others reported. Prophylactic antibiotics for PEG are recommended in both the ASGE (American Society of Gastroenterology) and BSG (British Society of Gastroenterology) guidelines [17, 18]; however, several PEG studies using the introducer method reported that it was unnecessary to use prophylactic antibiotics [19, 20]. With gastropexy, fistula formation is possible, even if we do not apply the pressure with the bumper, which is thought to contribute to the blood circulation defect due to excessive pressure. Gastropexy prevents peritonitis in the event of accidental removal of the gastrostomy tube before maturation of the gastrostomy tract. With gastropexy, the abdominal wall and stomach are fixed tightly and the gastrostomy tract matures rapidly and becomes more stable, which causes less-frequent damage to the gastrostomy tract at the time of exchanging the gastrostomy tube. Ascites is one of the contraindications of PEG, but Wejda *et al*. reported the safe placement of PEG in patients with ascites using gastropexy [21].

The main disadvantages of gastropexy are the complexity and long duration of the procedure. Other disadvantages are bleeding due to multiple punctures and the cost of the gastropexy device. Impairment of peristalsis by fixation is also a predictable complication, but we did not experience any definite cases of this.

Several studies have compared the pull-through and introducer techniques, and they all concluded that the introducer technique reduced the risk of peristomal infection, as in our study [10, 11]. They suggested that the reason why the introducer method reduced the risk of infection was that the gastrostomy tube did not pass through the mouth and pharyngeal bacterial flora; however in our small series, the infection rates of pull-PEG with gastropexy and introducer-PEG were almost the same. We are of the opinion that bacterial translocation is the most important factor in peristomal infection and inflammation, but other factors such as gastric juice leakage and local circulatory insufficiency by compression of the bumper are also related to peristomal inflammation. These results mean that, with gastropexy, the stoma infection rates were almost the same in pull- and direct PEG patients. Fixation of the stomal site itself seems to be very important in protecting against stomal infection. Although very small in number, our gastropexy pull-PEG group showed a very low rate of infection, while bleeding rate was lower and procedure duration was shorter than in the introducer-PEG group. Funada-style gastropexy is a simple and quick method, which can be performed successfully, regardless of experience. In our hospital, most PEG procedures are performed by Fellows without any severe complications. The Loop Fixture II advanced model of the Funada-type gastropexy device has been developed, which can be manipulated with a single hand, enabling us to perform gastropexy more quickly and easily.

This study is a single-centre, just retrospective, and non-randomized study. Therefore it has limitations. Many of studies that compare pull-PEG and introducer-PEG have emphasized that direct insertion from the abdominal wall prevents peristomal infection, but they did not assess the importance of gastropexy. Prospective study to compare pull-PEG and gastropexy pull-PEG is necessary to prove the importance of gastropexy. And a prospective randomized study to compare Funada-style gastropexy and T-fasteners is also necessary.

In conclusion, gastropexy seems desirable for the prevention of early complications of PEG, with either the pull- or introducer methods; however prospective study is required.
